# MSPFormer: An enhanced multi-scale and semantic-preserving transformer for sheep ownership identification in precision livestock farming

**DOI:** 10.1371/journal.pone.0349654

**Published:** 2026-07-21

**Authors:** Yaosheng Han, Chunmei Li, Xiangjie Huang, Qing Dong, Hao Wang

**Affiliations:** 1 School of Computer Technology and Application, Qinghai University, Xining, Qinghai, China; 2 Intelligent Computing and Application Laboratory of Qinghai Province, Qinghai University, Xining, Qinghai, China; Shandong Agricultural University, CHINA

## Abstract

Overgrazing is a major driver of grassland degradation on the Qinghai–Tibet Plateau, posing significant challenges for sustainable livestock management. To mitigate this issue, intelligent technologies that can effectively recognize and manage different herders’ sheep flocks are essential for achieving balanced grass–livestock management and reducing overgrazing. This study aims to develop a robust and efficient model for intelligent sheep ownership recognition by leveraging sheep back color features, facilitating scientific grazing management, and supporting herder conflict resolution. A dedicated dataset was constructed, capturing diverse color distributions under variable lighting and complex backgrounds. We propose MSPFormer, a multi-scale and semantic-preserving Transformer model, which builds upon Mask2Former by integrating three key modules: (1) an Atrous Spatial Pyramid Pooling (ASPP) module between the Pixel Decoder and Transformer Decoder to enhance multi-scale feature extraction; (2) a Dynamic Prompt Attention (DPA) module to improve semantic consistency; and (3) a Content-Aware ReAssembly of Features (CARAFE) upsampling module after the Transformer Decoder to optimize spatial detail recovery. Experimental results show that MSPFormer achieves superior segmentation performance, increasing the mIoU from 82.46% to 83.81% (a relative improvement of approximately 1.35%), the mF1-score from 90.08% to 90.92% (an improvement of approximately 0.84%), the mPrecision from 89.50% to 91.52% (an improvement of approximately 2.02%), and the mRecall from 90.74% to 90.92% (an improvement of approximately 0.18%). Validation on the public VOC2012 dataset and several small-sample subsets further demonstrates the model’s strong generalization capability and stability. This study provides an effective and intelligent solution for sheep ownership recognition, contributing to sustainable grazing management and conflict resolution among herders on the Qinghai–Tibet Plateau. Future work will focus on lightweighting the model through pruning and knowledge distillation to facilitate practical deployment.

## Introduction

Known as the “Roof of the World” and the “Water Tower of Asia” [1], the Qinghai-Tibet Plateau serves as a critical ecological security barrier globally [2], fulfilling dual roles of ecological conservation and livestock-based economic development [3]. Situated in an alpine and harsh natural environment [4], the region features vast natural grasslands and abundant, diverse livestock resources, making it a typical alpine pastoral area and a core development zone for animal husbandry [5]. The total grassland area on the Qinghai-Tibet Plateau is approximately 1.653 million square kilometers, with alpine meadows accounting for nearly 50% of the total [6–8]. These grasslands not only provide essential ecosystem services such as water conservation, biodiversity maintenance, and carbon cycle regulation [9,10], but also constitute the fundamental means of livelihood and production for local pastoral communities [11].

Due to its exposure to extreme climatic conditions—including low temperatures, aridity, and intense solar radiation—the Qinghai-Tibet Plateau ecosystem is highly fragile and particularly sensitive to both environmental and anthropogenic disturbances [12]. In recent decades, grassland degradation on the plateau has become increasingly severe [13]. Since the mid-20th century, under the combined influence of climate change and human activities, approximately one-third of the plateau’s grasslands have experienced varying degrees of degradation, with some regions showing extreme forms of deterioration, such as “black soil patches” [14–16]. This degradation not only constrains the sustainable development of animal husbandry but also poses potential threats to regional ecological security and social stability [17].

Grazing is the predominant land use practice on the Qinghai-Tibet Plateau and plays a critical role in maintaining the health and stability of grassland ecosystems through its intensity and management modes [18]. Traditionally, herders have relied on unfenced communal pastures to implement mobile grazing systems, forming a unique grassland resource-sharing mechanism and a nomadic production system. This approach has historically contributed to ecological balance and community cooperation in the region [19–21]. However, with the continuous expansion of animal husbandry and intensification of grazing pressure, the problem of grassland degradation induced by overgrazing has become increasingly prominent [22], manifestations include significant declines in vegetation cover, soil structure destruction, and accelerated water and soil erosion, leading to severe impairment of grassland ecosystem functions. Consequently, this threatens both regional ecological security and the sustainable development of animal husbandry [23–25].

Furthermore, unclear grassland tenure boundaries and the lack of standardized border management and effective coordination among herders have resulted in frequent mixing of sheep flocks from different herders on communal grasslands [26,27]. This phenomenon exacerbates competition and conflicts over grassland resources, significantly increases difficulties in individual livestock identification and ownership management [28,29], and thus constrains the potential for modern intelligent animal husbandry development.

In pastoral areas such as the Qinghai-Tibet Plateau, traditional methods of livestock ownership identification rely primarily on manual labeling techniques such as ear tags, spray paint, and shearing patterns. These methods are simple to operate and relatively low in cost, and thus have been widely adopted in small-scale grazing scenarios [30–32]. However, as the scale of livestock farming in pastoral areas continues to expand, these manual identification approaches are showing significant shortcomings [33,34]. Traditional methods depend on human visual inspection, which is time-consuming, subjective, and unable to meet the real-time management requirements of dynamic and complex environments [35,36]. Furthermore, the frequent maintenance and replacement of these identifiers increase labor and material costs, thereby restricting the modernization and intelligent development of grazing management [37]. In summary, traditional manual labeling methods exhibit poor visibility, high maintenance costs, and low identification efficiency in large-scale pastoral areas, seriously hindering precise livestock management and the advancement of smart pastoral practices. There is an urgent need to introduce automated and intelligent identification technologies to address the challenges of modern livestock management in the new era.

In recent years, with the rapid development of deep learning technologies, particularly in the field of computer vision, image-based semantic and instance segmentation methods have gradually become key technologies for automatic livestock identification and ownership determination [38–41]. Compared with traditional manual labeling methods, these automated recognition techniques not only enable high-precision segmentation of color-marked regions on the backs of sheep but also offer significant advantages, including non-invasiveness, high automation, and rapid real-time response [42,43], which greatly enhance the level of intelligent livestock management in pastoral areas.

Currently, various efficient models have emerged in the field of object detection and segmentation and have been widely applied in agriculture and animal husbandry [44]. The YOLO series, for example, is suitable for real-time monitoring due to its fast detection speed [45]; U-Net, with its strong detail-capturing capability, achieves high segmentation accuracy even in complex backgrounds [46]; SegFormer incorporates a Transformer-based architecture that enhances global context modeling [47], compensating for the limitations of traditional convolutional networks in multi-scale feature fusion; DeepLabv3 + leverages atrous convolution to improve the perception of features at different spatial scales, effectively addressing image segmentation tasks in complex environments [48]. The Mask2Former model innovatively combines the Transformer structure with a mask prediction mechanism, enabling more comprehensive capture of semantic and spatial features of targets [49], significantly improving segmentation performance in dense targets and regions with blurred boundaries, thereby demonstrating strong robustness and generalization ability.

Despite the success of these methods, existing Transformer-based architectures still face several limitations when applied to complex pastoral environments. Vision Transformer (ViT) primarily relies on global self-attention, which lacks explicit inductive bias for multi-scale feature modeling and often struggles to capture fine-grained local structures under occlusion and cluttered backgrounds. Swin Transformer introduces a hierarchical design with window-based self-attention to improve computational efficiency; however, its restricted window partitioning limits long-range cross-region semantic interaction, which is critical in densely clustered livestock scenarios.

In addition, CNN–Transformer hybrid architectures attempt to combine convolutional local feature extraction with global attention mechanisms. Nevertheless, they often suffer from insufficient semantic consistency across scales, particularly when dealing with blurred boundaries, illumination variations, and severe occlusions commonly observed in real-world grazing environments.

To address these limitations, the proposed MSPFormer integrates multi-scale feature modeling, semantic-preserving attention, and edge-aware feature reconstruction in a unified framework. Specifically, the model introduces feature-level multi-scale fusion via FPN and ASPP, enabling robust hierarchical representation; a Dynamic Prompt Attention (DPA) module to enhance semantic consistency and suppress background interference; and a CARAFE-based upsampling strategy to improve boundary detail preservation.

Beyond agricultural and livestock applications, similar challenges have also been extensively studied in high-resolution remote sensing image segmentation. For instance, a deep hybrid network combining DenseNet and U-Net was proposed for land cover semantic segmentation in high-spatial-resolution satellite images, where DenseNet is employed to enhance multi-scale feature extraction while the encoder–decoder structure with long-range skip connections preserves fine-grained spatial details [50]. This hybrid design has demonstrated strong robustness under complex imaging conditions such as illumination variations, occlusions, and heterogeneous backgrounds, highlighting the effectiveness of multi-scale feature fusion and low-level detail preservation in challenging segmentation scenarios. These findings further indicate that integrating complementary architectural components is a promising strategy for improving segmentation performance in complex natural environments.

Despite the great potential of deep learning-based image segmentation technologies for livestock automatic identification, the accurate segmentation of sheep back color regions in the complex and variable natural environment of the Qinghai-Tibet Plateau remains challenging. During grazing, sheep often cluster densely and move continuously, resulting in severe occlusion among individuals [51,52], which hinders the full visibility of color markings and affects recognition accuracy. Moreover, the lighting conditions in high-altitude areas are highly variable, with intense sunlight and sharp shadow contrasts that interfere with image quality [53], further reducing model perception capabilities. In addition, the boundaries of color markings are often blurred, and different herders may use similar colors [54], which increases the difficulty of distinguishing among them. Traditional segmentation models often lack sufficient capabilities in multi-scale feature fusion, contextual information modeling, and edge detail restoration, making them inadequate to meet the fine-grained segmentation requirements of highland pastoral areas. Furthermore, real-time monitoring of large-scale sheep herds imposes higher demands on the model’s computational efficiency and inference speed. Consequently, there is an urgent need to develop efficient segmentation models with strong multi-scale feature modeling, precise semantic understanding, and accurate edge detail restoration. Such advancements would enhance model adaptability and robustness in complex environments, advance smart animal husbandry, and support the integration of ecological protection with sustainable livestock production.

This study, focusing on the practical needs of smart animal husbandry management and ecological protection in the Qinghai-Tibet Plateau region, explores an effective technical pathway for recognizing sheep back color patterns and distinguishing herder ownership in complex natural backgrounds. To address the challenges posed by densely distributed sheep, severe inter-individual occlusion, and strong background interference in highland areas, we propose an improved instance segmentation model based on the Mask2Former framework—MSPFormer (Multi-Scale and Semantic-Preserving Transformer). Building upon the global modeling capabilities of the Transformer encoder, MSPFormer integrates structural optimization strategies such as multi-scale contextual perception, key semantic focus, and edge detail reconstruction, thereby significantly enhancing performance in multi-scale feature extraction, semantic preservation, and boundary restoration. By establishing a mapping relationship between sheep back color markings and herder ownership, this model enables automatic identification and precise attribution of sheep to different herders in mixed grazing scenarios, effectively overcoming the accuracy, efficiency, and scalability bottlenecks of traditional manual labeling methods. This approach not only improves instance segmentation performance in complex natural environments but also provides key technical support for the development of smart animal husbandry and ecological resource management in the Qinghai-Tibet Plateau. Moreover, it demonstrates good generalizability and potential for broader application in tasks such as wildlife monitoring and crop identification in various natural scenarios, offering significant scientific value and practical application prospects.

The main contributions of this study are as follows:

1. We constructed a sheep back color image dataset tailored to the natural grazing environment of the Qinghai-Tibet Plateau. The dataset comprises 5,490 high-quality annotated images, with 4,352 images for training and 1,138 for validation. Unlike existing livestock datasets that primarily focus on detection or coarse segmentation, this dataset is specifically designed for sheep ownership identification and provides fine-grained, pixel-level instance annotations of color-marked dorsal regions under real pastoral conditions. This dataset fully reflects the diversity of sheep back color patterns in the complex plateau environment and captures significant visual challenges such as occlusion, scale variation, and heterogeneous backgrounds, laying a solid foundation for model training and performance evaluation.
2. We propose an improved model, MSPFormer, based on the Mask2Former framework. MSPFormer integrates three key modules: Atrous Spatial Pyramid Pooling (ASPP), Dynamic Prompt Attention (DPA), and Content-Aware Reassembly of Features (CARAFE). The multi-scale strategy in MSPFormer is implemented at the feature hierarchy level rather than token-level or patch-level representation, enabling effective aggregation of contextual information across different spatial resolutions. This integration significantly enhances multi-scale feature perception, key semantic focus, and edge detail reconstruction capabilities, effectively improving the performance of sheep back color segmentation and herder ownership identification in complex plateau environments. Unlike conventional attention mechanisms such as SE or CBAM, the proposed Dynamic Prompt Attention (DPA) introduces task-aware semantic prompts to guide feature aggregation, thereby enhancing semantic consistency and reducing background interference in complex outdoor environments.
3. We conducted systematic experimental evaluations using a real-world dataset collected from pastoral areas of the Qinghai-Tibet Plateau. The results demonstrate that MSPFormer achieves outstanding performance in sheep back color segmentation and herder ownership identification tasks, outperforming several mainstream models. MSPFormer shows excellent generalization ability and broad application potential, providing crucial technical support for image segmentation in smart animal husbandry and ecological monitoring in complex natural scenarios.

## Materials and methods

### Dataset description

The dataset utilized in this study was collected through fieldwork conducted in Datong County and Guinan County of Qinghai Province. The research team captured images of sheep herds in pastoral areas, with a particular focus on the dorsal (back) color regions of the sheep, aiming to support instance segmentation and herder ownership identification tasks. Image acquisition was performed using a combination of high-resolution cameras and drones to comprehensively record the natural distribution of the sheep herds. Different from existing livestock datasets that mainly focus on detection, counting, or generic segmentation tasks, the proposed dataset is specifically designed for sheep ownership identification, which requires fine-grained instance-level segmentation of color-marked dorsal regions under real pastoral conditions. During data collection, special attention was paid to capturing various lighting conditions at different times of the day—including sunny and cloudy weather—and diverse backgrounds such as grasslands, rocks, and soil in the pastoral setting, introducing substantial visual complexity caused by occlusion among individuals, large scale variations, and background interference, thereby ensuring strong diversity and representativeness of the dataset. Video footage collected was processed using OpenCV to extract individual frames, with blurry or highly repetitive frames removed. As a result, a curated dataset comprising 5,490 images was established as the foundational sample set for this study.

For the data annotation stage, the research team employed the X-AnyLabeling tool to manually annotate the sheep back color regions frame by frame. Unlike most existing livestock datasets that rely on bounding-box annotations, each sheep dorsal region in our dataset was precisely delineated at the pixel level to generate high-quality instance segmentation annotations. These regions are commonly used in real pastoral production management to distinguish different herders’ sheep, featuring distinct segmentation characteristics and high practical application value. Based on the color distribution characteristics, the sheep back colors in the images were categorized into four classes: red, blue, yellow, and colourless. These colors not only represent the visual characteristics of the sheep’s back but also explicitly encode the ownership relationships among different herders, which is rarely addressed in existing public livestock datasets. Specifically, red, blue, and yellow labels represent sheep from different herders, while colourless indicates sheep not belonging to any particular herder. Table 1 presents the mapping between sheep back color and herder ownership, clarifying the practical meaning of each annotation label.

**Table 1:** Mapping between sheep back color and herder ownership.

Herder ID	Back Color	Description
Herder A	Red	The sheep back is marked in red to indicate ownership by Herder A.
Herder B	Blue	The sheep back is marked in blue to indicate ownership by Herder B.
Herder C	Yellow	The sheep back is marked in yellow to indicate ownership by Herder C.
Unassigned	Colourless	The sheep back is unmarked, and ownership has not yet been determined.

**Fig 1:**
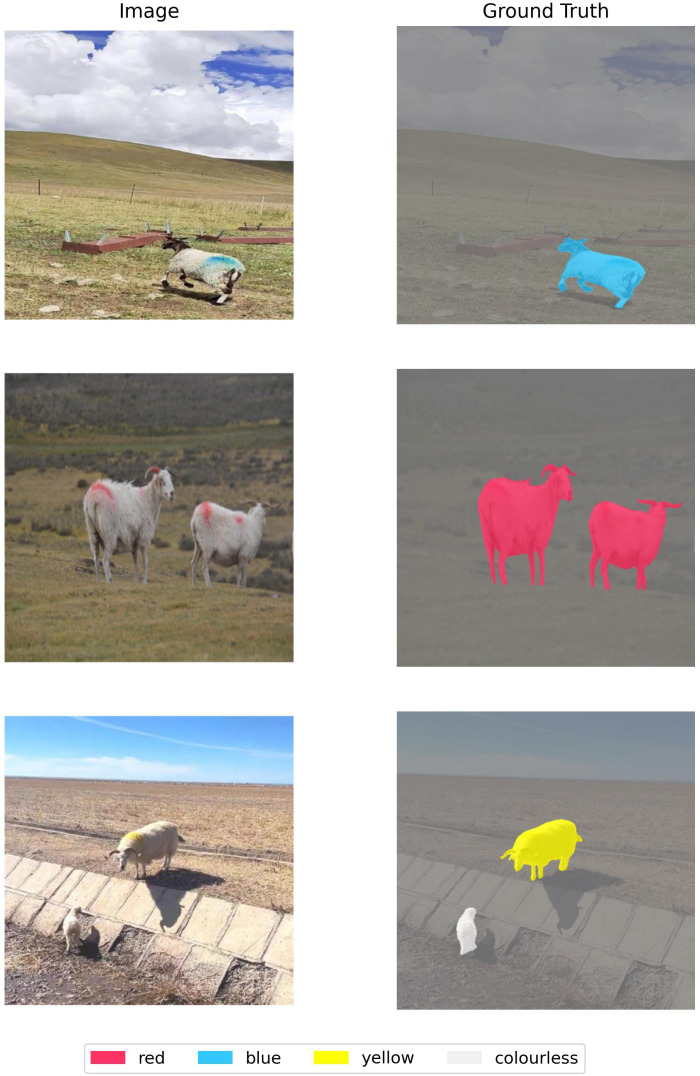
Example images of the four types of color-coded labels.

The number of sheep varies across images, and the annotation team meticulously delineated the back of each sheep to generate corresponding instance labels. Fig 1 provides example images illustrating the four types of color-coded labels, including a comparison between the original images and their annotated counterparts.

**Fig 2:**
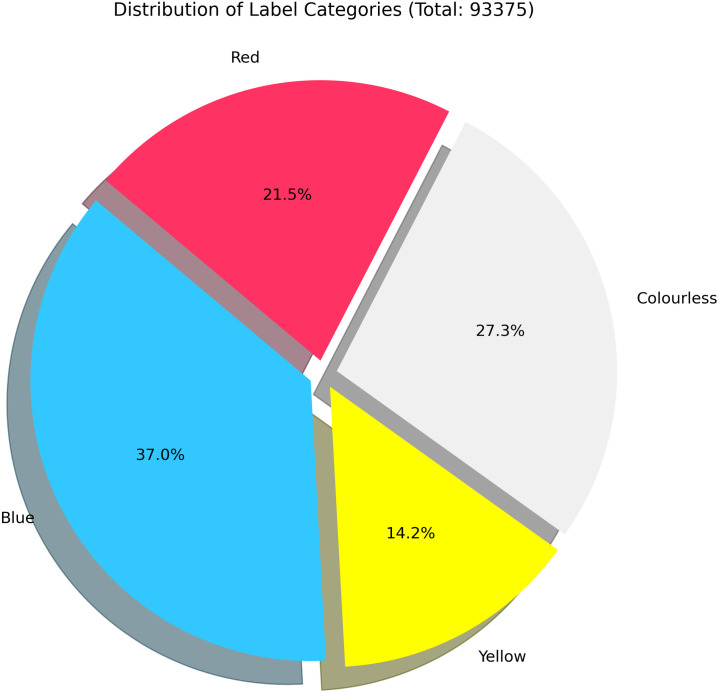
Distribution of label counts across different categories.

After annotation, a total of 93,375 valid labels were obtained, including 34,525 labels in the blue category, 13,303 in the yellow category, 25,488 in the colorless category, and 20,059 in the red category. The resulting dataset therefore provides a high density of fine-grained, instance-level annotations under real-world pastoral conditions, enabling rigorous evaluation of segmentation performance for ownership identification tasks. Fig 2 illustrates the distribution of label counts for each category. Overall, the dataset demonstrates a relatively balanced distribution across categories. Although slight proportional differences exist, the dataset exhibits good representativeness and reasonable distribution after cleaning and optimization.

To ensure the scientific rigor of training and evaluation, the dataset was divided into a training set and a validation set at a 4:1 ratio. Specifically, the training set contains 4,392 images, while the validation set includes 1,098 images. Table 2 presents the detailed distribution of different label categories within the training and validation sets.

**Table 2:** Label distribution across the training and validation sets.

Label Category	Train	Val	Total
Red	15,942	4,117	20,059
Blue	27,452	7,073	34,525
Yellow	10,579	2,724	13,303
Colourless	20,258	5,230	25,488
All labels	74,231	19,144	93,375

As shown in Table 2, the label distribution across the training and validation sets is consistent among the different categories, adhering closely to the 4:1 split ratio. This balanced distribution ensures that the model can effectively learn features associated with different color-coded labels during the training phase, while also providing a representative and stable evaluation during the validation phase. Although the red category shows a slight deviation between the two subsets, the overall distribution remains consistent. This consistency enhances the model’s ability to generalize across different categories and improves its robustness, thereby increasing its practical value in real-world applications.

## Model architecture

### Transformer-based image segmentation model: Mask2Former and its architectural improvements

Image segmentation, a fundamental task in computer vision, aims to achieve precise recognition and division of different regions within an image [55]. In complex natural scenes, such as the segmentation of sheep back colors on the Tibetan Plateau, images exhibit high diversity and intricate details, posing significant challenges for models in terms of context-awareness and multi-scale information fusion. The dense distribution of sheep, severe individual occlusion, blurred boundaries of color markings, and interference from complex backgrounds further complicate the segmentation process. Effectively integrating multi-scale features and accurately capturing fine-grained regions are key to achieving high-quality segmentation.

Mask2Former is a state-of-the-art image segmentation method developed recently, based on the Transformer architecture. It innovatively introduces a mask classification strategy, enabling a unified framework for semantic, instance, and panoptic segmentation tasks [56]. Traditional segmentation approaches mainly rely on pixel-wise classification, predicting categories for each pixel and subsequently using complex post-processing to obtain instance segmentation, which is both cumbersome and inefficient. Mask2Former, by contrast, directly predicts binary masks and their categories through dynamically generated mask queries, greatly simplifying the segmentation pipeline and improving model flexibility and inference efficiency. The architecture comprises three main modules: a deep convolutional backbone (e.g., ResNet series) that extracts multi-scale and multi-level visual features, capturing both detailed textures and semantic information; a Pixel Decoder that fuses and refines these multi-scale features to enhance spatial resolution and detail representation, thereby improving accuracy for complex boundaries and fine-grained objects; and a Transformer Decoder that leverages multi-head self-attention to fully exploit global context, enabling deep interaction between mask queries and pixel features, enhancing long-range dependency modeling, and accurately capturing object shapes and semantic boundaries. The mask classification mechanism enhances the model’s ability to distinguish multiple similar instances in complex scenes, while effectively handling occlusions and blurred boundaries, demonstrating excellent robustness and generalization. Mask2Former adapts well to diverse image content and complex backgrounds, combining global semantic perception with fine-grained instance recognition, thus meeting the demands of various segmentation tasks. Overall, by integrating deep multi-scale feature fusion, Transformer-based global context modeling, and an innovative mask classification strategy, Mask2Former overcomes limitations of traditional segmentation models in detail restoration and instance separation, establishing itself as a representative and broadly applicable advanced framework in visual segmentation.

Despite its excellent performance, the original Mask2Former architecture has limitations when tackling specific complex scenarios, particularly in the challenging task of sheep back color segmentation in high-altitude pastoral areas, as addressed in this study. This task is characterized by ambiguous object boundaries, uneven color distributions, and complex background interference, significantly increasing the demands for multi-scale information modeling and detail recovery. First, the original model’s multi-scale semantic fusion is limited, making it difficult to effectively combine shallow detailed textures with deep semantic features, which reduces its ability to represent fine structures and small objects. Second, the Pixel Decoder uses a fixed upsampling strategy, which risks information loss during spatial reconstruction, affecting accurate restoration of object edges and local details. Moreover, although the Transformer Decoder provides global modeling capabilities, its attention mechanism lacks dynamic adaptability. When confronted with dense occlusions, cluttered backgrounds, or high similarity among individual sheep, it struggles to precisely focus on key regions, leading to decreased discriminability in mask prediction. Therefore, to improve Mask2Former’s segmentation performance in complex natural environments, it is necessary to introduce more flexible feature fusion mechanisms, higher-fidelity upsampling modules, and more selective dynamic attention strategies to enhance its perception of detailed areas and semantic boundaries, thereby achieving more accurate and robust segmentation results.

To further improve the image segmentation performance of Mask2Former in complex scenarios—particularly addressing the challenges posed by diverse and detailed sheep back color distribution, occlusion, and background interference in high-altitude pastoral areas—this study introduces three architectural improvements at critical stages of its original framework. These enhancements systematically strengthen the model’s representational capability and robustness from three perspectives: multi-scale context modeling, attention-guided mechanisms, and high-quality feature reconstruction.

First, an Atrous Spatial Pyramid Pooling (ASPP) module is introduced between the Backbone and the Pixel Decoder.The ASPP module employs parallel atrous convolutions with varying sampling rates to capture multi-scale contextual information, effectively enhancing the joint modeling of local and global semantics for mid- and low-level features [57]. This alleviates the semantic loss issue in the multi-scale fusion process of the original framework. By significantly expanding the receptive field, the introduction of ASPP enables the network to better recognize target regions with complex distributions and varying sizes.

Second, a Dynamic Prompt Attention (DPA) module is embedded between the output of the Pixel Decoder and the Transformer Decoder.The DPA module introduces a set of learnable dynamic prompt tokens [58] to guide the attention mechanism toward more discriminative target regions. During the self-attention process, the DPA module by dynamically adjusting the importance distribution across different feature regions, enhancing the ability to capture fine-grained features. This is particularly effective in handling heavily occluded, boundary-blurred, or color-similar targets, thereby improving recognition accuracy.

Finally, a Content-Aware ReAssembly of Features (CARAFE) module [59] is introduced at the output stage of the Transformer Decoder to replace traditional upsampling operations. Traditional bilinear or transposed convolutions often result in blurred details or artifacts during feature reconstruction [60]. In contrast, CARAFE adaptively generates reassembly weights based on local content, achieving high-fidelity restoration of spatial structural information. This significantly improves the edge quality and spatial consistency of the predicted masks, making it particularly suitable for fine-grained segmentation tasks involving complex boundaries and small objects.

The three improvement modules target the critical stages of feature extraction, feature interaction, and feature reconstruction, forming a synergistic optimization strategy from input to output. These enhancements maintain the general applicability of the Mask2Former architecture while significantly improving its adaptability and segmentation accuracy in real-world complex scenarios.The following sections will provide a detailed explanation and analysis of the structural design, embedding methods, and practical effects of these modules.

**Fig 3:**
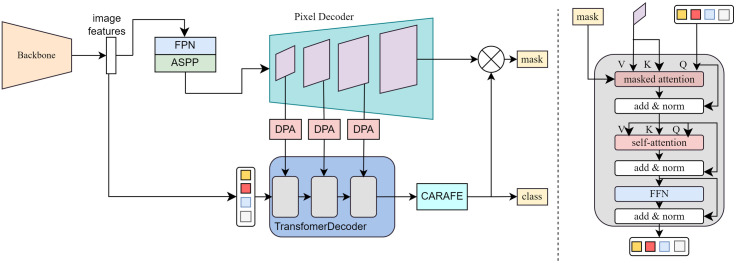
Schematic Diagram of the Improved Mask2Former Module Architecture.

Fig 3 illustrates the detailed structure of the improved Mask2Former architecture. The model first extracts multi-level foundational features from the input image using a backbone network, which serves as the basis for subsequent processing. To further enhance the representation capability of multi-scale features, a Feature Pyramid Network (FPN) is introduced following the backbone output, enabling effective fusion of features at different levels and contextual supplementation [61]. To address the limited multi-scale feature fusion ability in the original architecture, an Atrous Spatial Pyramid Pooling (ASPP) module is inserted between the FPN output and the Pixel Decoder. This module captures rich contextual information through parallel multi-scale atrous convolutions, significantly improving the joint perception of local and global semantics in mid- and low-level features. The Pixel Decoder further decodes the fused features to extract detailed multi-scale spatial semantic information. To enhance the Transformer Decoder’s focus on critical regions, a Dynamic Prompt Attention (DPA) module is introduced between the Pixel Decoder output and Transformer Decoder input. The DPA module guides the attention mechanism with learnable dynamic prompt vectors to dynamically allocate attention weights, markedly improving the recognition ability for occluded, boundary-ambiguous, and color-similar targets in complex scenes. The Transformer Decoder performs mask and class predictions based on the enhanced attention features, achieving global information interaction and detail extraction via self-attention mechanisms. Finally, to mitigate detail loss and artifacts commonly caused by traditional upsampling methods, a content-aware reassembly module (CARAFE) is appended after the Transformer Decoder output. CARAFE adaptively generates reassembly weights based on local content, enabling high-quality spatial feature reconstruction, which effectively enhances the sharpness and spatial consistency of predicted mask boundaries.

### Differences from existing transformer architectures

Existing Vision Transformer (ViT) models rely on global self-attention, which lacks explicit multi-scale inductive bias and struggles to capture fine-grained local structures in complex outdoor environments with occlusion and background clutter. Although Swin Transformer introduces a hierarchical architecture with window-based self-attention to improve computational efficiency, its limited receptive field within fixed windows restricts cross-region semantic interaction, which is critical for densely clustered livestock scenes.

Hybrid CNN–Transformer architectures attempt to combine convolutional local feature extraction with global attention mechanisms; however, they often fail to maintain consistent semantic representation across scales, especially under conditions of severe occlusion, illumination variation, and boundary ambiguity.

In contrast, the proposed MSPFormer addresses these limitations by integrating feature-level multi-scale modeling, semantic-preserving attention, and edge-aware feature reconstruction. Specifically, multi-scale contextual information is captured via FPN and ASPP, semantic consistency is enhanced through Dynamic Prompt Attention (DPA), and fine boundary details are refined using CARAFE-based upsampling.

### Multi-scale context awareness: Integration of the ASPP module

In the task of segmenting sheep back color regions within complex natural scenes, the model faces significant challenges due to scale variations and background interference. Although the Pixel Decoder in the original Mask2Former possesses a certain capability for spatial semantic integration, its ability to model long-range context is limited, resulting in insufficient capture of fine-grained semantic features in small targets and boundary regions. To address this, the present study employs a lightweight Feature Pyramid Network (FPN), to first fuse multi-scale features, followed by an Atrous Spatial Pyramid Pooling (ASPP) module to enhance the model’s multi-scale context awareness. The multi-scale feature fusion in this design operates at the feature hierarchy level, rather than token-level or patch-level representation, enabling effective aggregation of semantic information across different spatial resolutions.

**Fig 4:**
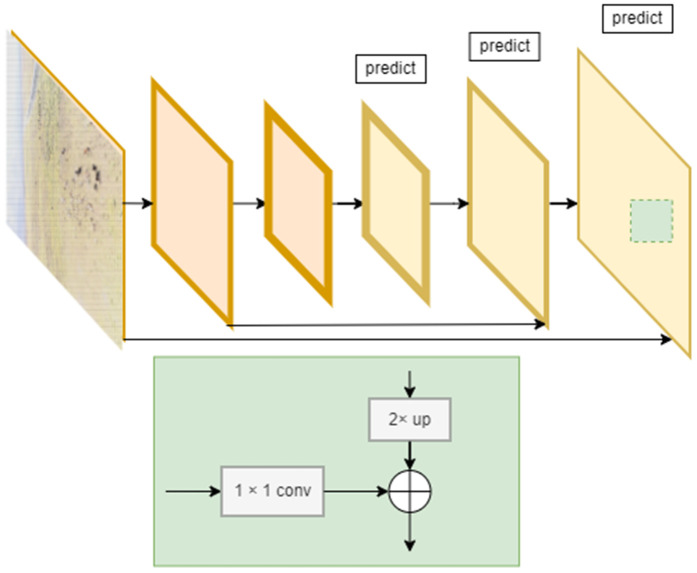
Schematic diagram of the lightweight Feature Pyramid Network (FPN) architecture.

Specifically, the FPN module performs a top-down feature fusion to unify semantic information across different resolutions, ensuring semantic consistency while enriching spatial detail representation. This process provides the subsequent ASPP module with multi-resolution and semantically rich input features. Fig 4 illustrates the top-down fusion process of different scale features in the FPN, highlighting the integration mechanism across multiple feature levels.

**Fig 5:**
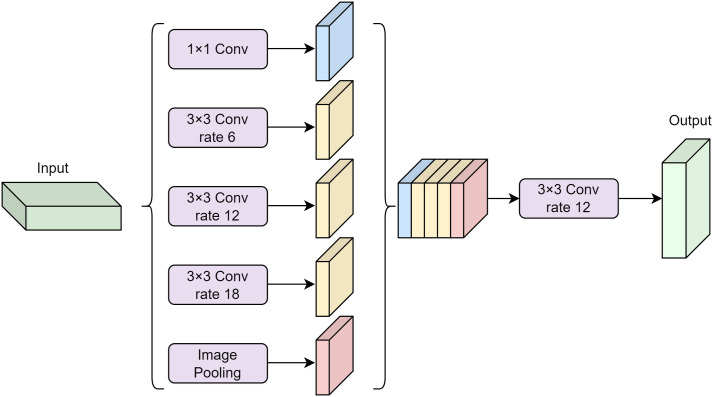
Schematic diagram of the Atrous Spatial Pyramid Pooling (ASPP) module.

The Atrous Spatial Pyramid Pooling (ASPP) module consists of five parallel branches, as illustrated in Fig 5: a 1×1 convolution branch, three 3×3 atrous convolution branches with different dilation rates (r = 6, 12, 18), and a global average pooling branch. Each branch is designed to capture contextual information at specific scales. Compared with single-scale convolutional or attention-based modules, ASPP explicitly expands the receptive field through multi-dilation parallel branches, enabling more robust modeling of scale variations and improving segmentation performance in cluttered and occluded environments. The outputs of these branches are concatenated along the channel dimension and subsequently fused through a 1×1 convolution to generate a feature map with rich multi-scale semantic representation.

This figure illustrates the five parallel branches of the ASPP module and their fusion process, including multi-scale atrous convolutions and global pooling. Atrous convolution enlarges the receptive field by inserting spaces (dilations) between kernel elements without increasing the number of parameters. Its computation can be expressed as:


$$\begin{array}{c} y[i]=\sum_{k}x[i+r\cdot k]\cdot w[k] \end{array}$$ (1)

Here, *x*[*i*] denotes the input feature, *w*[*k*] represents the convolution kernel weight, and *r* is the dilation rate. This design enhances the model’s ability to capture long-range contextual information and improves its recognition of multi-scale objects.

The features fused by the ASPP module are first aligned along the channel dimension and then fed into the Transformer Decoder for final mask prediction. The enriched multi-scale features produced by the ASPP module provide stronger contextual priors for the subsequent Transformer Decoder, improving its ability to distinguish fine-grained boundary regions and small-scale objects. This module demonstrates excellent performance in modeling the spatial variability and global semantic relationships of sheep back color regions, particularly improving small object recognition and edge detail restoration.

By combining the FPN-based multi-scale feature fusion with the ASPP module, the model significantly enhances its capability to perceive contextual information at varying scales, effectively enriching semantic feature representation and preserving spatial details. This enables better adaptation to the segmentation demands in complex natural environments.

### Dynamic feature guidance mechanism: Introduction of the DPA module

In conventional Transformer-based segmentation architectures, query vectors are typically initialized in a fixed manner, lacking the ability to adaptively represent image content [62]. This static design often leads to imprecise attention and blurred object boundaries when handling complex backgrounds, small targets, or semantically ambiguous adjacent regions, thereby limiting the model’s fine-grained segmentation capability. To address this limitation, this study proposes a Dynamic Prompt Attention (DPA) module. By introducing a content-driven dynamic prompt generation mechanism [63] and integrating multi-level memory features for enhanced attention, the module guides the model to focus on discriminative regions effectively, thereby significantly improving the accuracy and robustness of target modeling. Unlike conventional attention mechanisms, the proposed DPA explicitly preserves semantic consistency across hierarchical features by using dynamic prompts to guide query generation, ensuring that discriminative object-related semantics are maintained during feature interaction.

**Fig 6:**
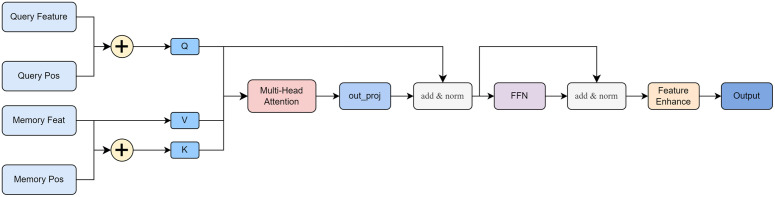
Structure diagram of the DPA module.

Different from channel/spatial attention mechanisms such as SE and CBAM, which perform static feature reweighting, DPA introduces dynamic semantic prompts that are conditioned on image content, enabling adaptive query refinement rather than fixed feature modulation. The DPA module mainly consists of two subcomponents: the Dynamic Prompt Generator and the Prompt-Guided Attention Fusion module. Its overall workflow is illustrated in Fig 6.

First, the DPA module receives multi-scale features from the output of the Pixel Decoder as memory features. Combined with pre-set or learnable positional encoding, it generates context-aware prompt vectors. This process leverages linear mapping and positional encoding to allow the prompt to dynamically reflect the semantic structure of the image. These prompts are not fixed learnable embeddings, but are dynamically generated based on input feature context, allowing the model to adaptively focus on discriminative regions under varying environmental conditions such as occlusion, illumination changes, and inter-class similarity. The generated prompt vectors, together with the initial queries, are fed into a multi-head attention module. Through interactive computation, the model captures the focus areas and their importance across different memory levels. Finally, the attention output is fused and enhanced through residual connections, normalization, and a feed-forward network to further strengthen feature representation capabilities.

### Detailed Computation Process

**(1) Positional Encoding Fusion and Linear Mapping.** Given the input query features $𝐐\in {\mathbb{R}}^{B\times N\times C}$, positional encoding ${𝐏}_{q}\in {\mathbb{R}}^{B\times N\times C}$, multi-scale memory features ${𝐌}_{l}\in {\mathbb{R}}^{B\times HW\times C}$ and their positional encoding ${𝐏}_{l}\in {\mathbb{R}}^{B\times HW\times C}$, positional fusion and linear mapping are first performed to generate new attention inputs:


$$\begin{array}{c} \hat{𝐐}=W_{q}(𝐐+{𝐏}_{q}),\;\hat{𝐊}=W_{k}({𝐌}_{l}+{𝐏}_{l}),\;\hat{𝐕}=W_{v}{𝐌}_{l} \end{array}$$ (2)

where **Q** is the original query, ${𝐌}_{l}$ is the memory feature at level *l*, ${𝐏}_{q}$ and ${𝐏}_{l}$ are the corresponding positional encodings, and $W_{q},W_{k},W_{v}\in {\mathbb{R}}^{C\times C}$ are learnable *l*inear projection matrices.

**(2) Multi-Head Attention Interaction** The features $\hat{𝐐},\hat{𝐊},\hat{𝐕}$ are split into *h* heads, each with dimension *d* = *C* / *h*. The attention output is computed as:


$$\begin{array}{c} Attention({\hat{𝐐}}_{h},{\hat{𝐊}}_{h},{\hat{𝐕}}_{h})=Softmax\left(\frac{{\hat{𝐐}}_{h}{\hat{𝐊}}_{h}^{⊤}}{\sqrt{d}}\right){\hat{𝐕}}_{h} \end{array}$$ (3)

The outputs from all heads are concatenated and projected using $W_{o}\in {\mathbb{R}}^{C\times C}$:


$$\begin{array}{c} AttnOut=W_{o}\cdot Concat({head}_{1},\ldots ,{head}_{h}) \end{array}$$ (4)

**(3) Residual Connection and Feature Enhancement** The attention output is added to the original query feature via residual connection and normalization, followed by a feed-forward network (FFN) to enhance feature expression:


$$\begin{array}{c} {𝐐}_{attn}=LayerNorm(𝐐+AttnOut) \end{array}$$ (5)

$$\begin{array}{c} {𝐐}_{ffn}=LayerNorm\left({𝐐}_{attn}+FFN({𝐐}_{attn})\right) \end{array}$$ (6)

where:


$$\begin{array}{c} FFN(x)=W_{2}\cdot ReLU(W_{1}x) \end{array}$$ (7)

with $W_{1}\in {\mathbb{R}}^{C\times 4C}$ and $W_{2}\in {\mathbb{R}}^{4C\times C}$.

**(4) Activation Output.** The result is then passed through a nonlinear activation module to further enhance feature discriminability:


$$\begin{array}{c} {𝐐}_{out}=ReLU(W_{e}{𝐐}_{ffn}) \end{array}$$ (8)

where $W_{e}$ is a learnable linear transformation weight matrix that maps the features for improved expression.

This process is independently performed at each memory feature level, ultimately producing enhanced query vectors that integrate multiple semantic hierarchies, providing more discriminative target representations for the subsequent Transformer decoder.

The DPA module, while maintaining computational efficiency, significantly improves the expressiveness of feature interactions by introducing dynamic prompts and prompt-guided attention fusion. Compared to fixed queries or global attention in conventional Transformers, DPA demonstrates stronger contextual awareness, enabling adaptive focus on target regions and enhancing robustness against complex backgrounds and occlusions. Furthermore, the module achieves effective cross-level feature fusion, avoiding shallow feature interference with deep semantic representation and improving multi-scale integration. The fused query vectors, enhanced by residual connections and nonlinear activation, exhibit stronger target discriminability, making them particularly suitable for small targets and fine-grained regions. In addition, the DPA structure is clear, with moderate parameter complexity, easy to integrate into existing Transformer decoders without major modifications, and exhibits excellent scalability.

### High-quality upsampling: Integrating the CARAFE module

Upsampling is a crucial component in semantic segmentation networks. It primarily restores the spatial resolution of feature maps and reconstructs fine details. During the encoding stage, the resolution of the feature maps is progressively reduced to extract rich semantic information. The upsampling stage then restores these low-resolution features to the size of the input image, enabling pixel-level precise segmentation. Traditional upsampling methods, such as bilinear interpolation, use fixed weights and therefore lack adaptability to input content, which often results in blurred edge details. While transposed convolution can learn upsampling weights, it suffers from limitations imposed by fixed kernel sizes and may produce checkerboard artifacts, which negatively affects the continuity and accuracy of segmentation results. To address these limitations, content-aware upsampling methods have emerged, leveraging dynamically generated convolution kernels to adaptively reassemble local features and overcome the drawbacks of traditional methods. Among these, the CARAFE (Content-Aware ReAssembly of FEatures) module stands out, employing a lightweight structure to implement dynamic kernel prediction and content-aware feature reassembly. This significantly enhances the detail reconstruction and edge preservation in the upsampling stage. In this study, the CARAFE module is integrated into the Mask2Former network to improve fine-grained segmentation performance in the challenging task of sheep back color segmentation under complex backgrounds. This design ensures that feature upsampling is performed at a semantically enriched stage of the Transformer decoder, where global contextual information has already been established, allowing CARAFE to focus on restoring spatial details while preserving semantic consistency.

The CARAFE module is a lightweight and flexible upsampling approach that achieves high-quality feature reassembly through content-aware dynamic convolution kernels. It consists of two main sub-modules: the Kernel Prediction Module and the Content-Aware Reassembly Module [64]. Specifically, the Kernel Prediction Module first generates local adaptive convolution kernel weights based on the input features. These weights are then utilized in the Content-Aware Reassembly Module to perform weighted reassembly of the input feature maps, thereby achieving high-quality upsampling. From a multi-scale perspective, CARAFE complements the feature-level fusion performed by FPN and ASPP by enhancing spatial resolution recovery, forming a complete hierarchical representation pipeline from coarse semantic aggregation to fine-grained detail reconstruction. Due to its simple design, high computational efficiency, and ease of integration into existing segmentation frameworks, the CARAFE module represents an effective solution for high-quality upsampling.

**Kernel Prediction Module.** This module is responsible for generating dynamic convolution kernels corresponding to each spatial location. The process consists of the following steps:

First, the input feature map $X\in {\mathbb{R}}^{B\times C\times H\times W}$ undergoes a channel compression operation to reduce computational costs.

Then, a convolutional operation is applied to predict the upsampling kernel weights for each pixel position, resulting in a weight tensor of shape ${\mathbb{R}}^{B\times (k^{2}\cdot r^{2})\times H\times W}$, where *k* denotes the kernel size and *r* represents the upsampling ratio.

Finally, this weight tensor is upsampled to match the resolution of the target output map, preparing it for the subsequent feature reassembly stage.

**Content-Aware Reassembly Module.** This module utilizes the dynamically generated kernel weights from the previous stage to reassemble the original feature map. Specifically, for each position in the output feature map, it centers on the corresponding position in the original feature map, extracts a local k×k region, and performs a weighted summation using the dynamic kernel weights to achieve content-aware feature fusion. Unlike transposed convolution with fixed kernels, this approach can adapt the reassembly of each position based on image content, thus preserving edge and structural details more effectively.

The process can be summarized as:

- Generating convolution kernels in a content-aware manner;
- Reassembling features spatially in a center-focused way;
- Achieving high-fidelity upsampling from low to high resolution.

**Fig 7:**
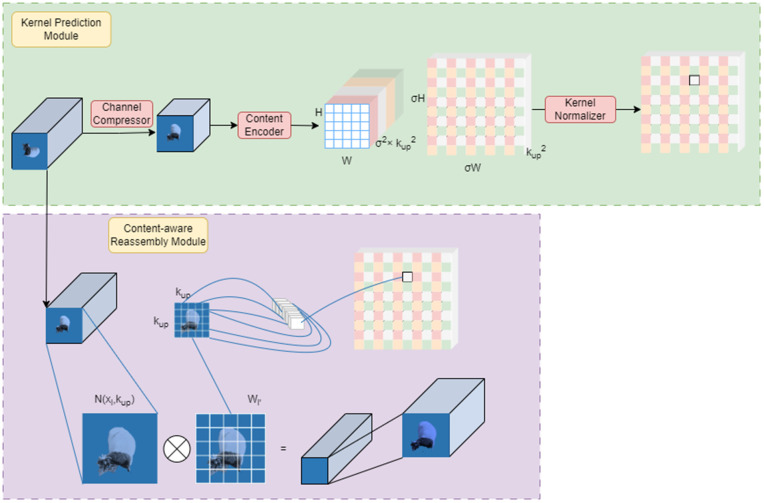
Schematic Diagram of the Content-Aware Reassembly Module (CARAFE).

The CARAFE module features a highly modular design that does not rely on additional prior information and can be easily integrated into various semantic segmentation networks. Its structural diagram is illustrated in Fig 7, which clearly demonstrates the two-stage process of kernel generation and content-aware feature reassembly.

Let the input feature map be:


$$\begin{array}{c} X\in {\mathbb{R}}^{B\times C\times H\times W} \end{array}$$ (9)

where *B* denotes the batch size, *C* represents the number of channels, and *H*, *W* are the spatial dimensions. The goal of CARAFE is to upsample this feature map to:


$$\begin{array}{c} \hat{X}\in {\mathbb{R}}^{B\times C\times \sigma H\times \sigma W} \end{array}$$ (10)

where σ denotes the upsampling factor.

### Kernel prediction module

As illustrated in the lower part of Fig 7, the Kernel Prediction Module comprises three steps:

1. **Channel Compressor**

A 1×1 convolution is used to reduce the number of channels from *C* to $C_{m}$, thereby decreasing subsequent computational complexity:


$$\begin{array}{c} X_{cc}={\text{Conv}}_{1\times 1}(X)\in {\mathbb{R}}^{B\times C_{m}\times H\times W} \end{array}$$ (11)

2. C**ontent Encoder**

A convolution with a larger receptive field (e.g., $k_{enc}=3$ or 5) is applied to extract the local context around each position and generate learnable reassembly kernels:


$$\begin{array}{c} K={\text{Conv}}_{k_{enc}\times k_{enc}}(X_{cc})\in {\mathbb{R}}^{B\times (\sigma^{2}\cdot k_{up}^{2})\times H\times W} \end{array}$$ (12)

where $k_{up}$ denotes the kernel size used for reassembly, and $\sigma^{2}$ indicates that each input position corresponds to σ×σ output positions. Therefore, $\sigma^{2}\cdot k_{up}^{2}$ reassembly weights must be predicted for each position.

3. **Kernel Normalizer**

The $k_{up}\times k_{up}$ convolution kernel predicted for each position is normalized using the softmax function to ensure that the sum of the weights equals one:


$$\begin{array}{c} {\hat{K}}^{\left(i,j\right)}=\text{Softmax}(K^{\left(i,j\right)})\in {\mathbb{R}}^{\sigma^{2}\times k_{up}^{2}} \end{array}$$ (13)

This yields a weight map for feature reassembly.

### Content-aware reassembly module

As shown in the upper part of Fig 7, this module receives the original feature map *X* and its corresponding weights *K* to perform feature reconstruction. For each position $\left(i^{\prime },j^{\prime }\right)$ in the upsampled output, the corresponding center in the original feature map is:


$$\begin{array}{c} (i,j)=\left(\left\lfloor \frac{i^{\prime }}{\sigma }\right\rfloor ,\left\lfloor \frac{j^{\prime }}{\sigma }\right\rfloor \right) \end{array}$$ (14)

A weighted reassembly is then performed on the $k_{up}\times k_{up}$ neighborhood 𝒩(i,j):


$$\begin{array}{c} {\hat{X}}_{b,c,i^{\prime },j^{\prime }}=\sum_{\left(u,v)\in 𝒩(i,j\right)}K_{(i^{\prime },j^{\prime }),u,v}^{\left(i,j\right)}\cdot X_{b,c,u,v} \end{array}$$ (15)

where $K_{(i^{\prime },j^{\prime }),u,v}^{\left(i,j\right)}$ denotes the reassembly kernel obtained from the content encoder after softmax normalization, 𝒩(i,j) is the $k_{up}\times k_{up}$ neighborhood centered at input position (*i*,*j*), and $\hat{X}$ is the upsampled output where each position is a weighted sum of features within the local neighborhood.

Essentially, this operation performs content-aware, dynamically weighted local region reconstruction, thereby achieving upsampling.

To achieve high-quality spatial detail recovery while maintaining high-level semantic consistency, the CARAFE module is integrated after the Transformer Decoder. The Transformer Decoder excels at capturing global semantic information, making it an ideal foundation for structural reconstruction. CARAFE, with its content-adaptive design, performs upsampling at this stage, which helps reconstruct fine spatial details without compromising semantic consistency. Moreover, by combining the outputs of the Pixel Decoder with features enhanced by the Transformer Decoder, this module achieves collaborative fusion of local and global information, significantly improving the clarity and accuracy of segmentation boundaries.

In semantic segmentation tasks, CARAFE demonstrates excellent adaptability, particularly in high-resolution image processing under complex backgrounds. Its core advantages include: (1) strong capability for preserving edges that effectively restores the contour details of the sheep’s back, thereby enhancing segmentation quality; (2) fine-grained representation for small targets, mitigating information loss that often occurs in conventional upsampling methods; and (3) lightweight structure and low computational overhead, making it suitable for integration with attention modules like DPA, thus further improving model representation and generalization capabilities. Therefore, CARAFE is not only well-suited for the sheep back color segmentation task in this study but also shows potential for broader application in other semantic segmentation scenarios. Unlike traditional interpolation-based upsampling methods, CARAFE learns content-dependent reassembly kernels, enabling adaptive reconstruction conditioned on local semantic context rather than fixed geometric rules.

## Experiments and analysis

### Experimental setup

#### Experimental environment.

All experiments in this study were conducted on the Linux Ubuntu 22.04 operating system. The development environment was implemented using Python 3.10.16, and the deep learning framework was PyTorch 2.0.1 with CUDA 11.7 (cu117). Supporting libraries included TorchVision 0.15.2, OpenCV 4.10.0, and MMEngine 0.10.7. The hardware configuration comprised an NVIDIA GeForce RTX 4090 GPU, a high-performance 16-core AMD EPYC 9354 CPU, and sufficient RAM to ensure efficient and stable model training and inference.

Regarding the compilation environment, PyTorch was built with GCC 9.3, and integrated several performance optimization libraries, such as Intel MKL, CuDNN 8.5, and AVX2 instruction sets, which significantly enhanced computational efficiency and resource utilization.

To ensure the reproducibility of experimental results, all dependencies were strictly version-controlled according to the aforementioned configurations. Additionally, a virtual environment was used to isolate the experimental environment, effectively preventing external interference and ensuring the consistency and stability of the experiments.

### Model parameters and training configuration

In this study, model training and evaluation were implemented using PyTorch in conjunction with MMEngine and MMSegmentation. Input images were preprocessed through fixed-size cropping (512×512), mean normalization, and standard deviation normalization. To enhance the generalization ability of the model, data augmentation strategies—including random cropping (scale range: 0.5 to 2.0), random flipping (probability: 0.5), and photometric distortion—were applied.

During model training, the batch size was set to 8, with a maximum of 160,000 iterations and validation performed every 500 iterations. The AdamW [65] optimizer was adopted, with an initial learning rate of 0.0002 and a weight decay coefficient of 0.05. The learning rate scheduling strategy employed a polynomial decay (PolyLR) [66] with a power parameter of 0.9. The loss function was a combination of cross-entropy loss and Dice loss [67], where the weight of the Dice loss was set to 5.0. To ensure the reproducibility of experimental results, the random seed was fixed at 0.

For model design, a pre-trained ResNet-50 was utilized as the backbone network, in combination with an FPN neck and a Mask2Former decoder head for the segmentation task. Leveraging the high-performance hardware environment, along with multiple optimization strategies and data augmentation techniques, ensured efficient and stable model training while improving the segmentation performance.

### Evaluation metrics

To comprehensively evaluate the performance of the models for the task of sheep back color segmentation, four commonly used pixel-level evaluation metrics were employed: Mean Intersection over Union (mIoU), Mean Precision (mPrecision), Mean Recall (mRecall), and F1 Score. These metrics assess the classification accuracy and boundary discrimination capability of the model for the target regions.

Specifically, mIoU is used to evaluate the overall segmentation accuracy across all color categories, reflecting the model’s ability to correctly segment sheep back color regions under complex backgrounds. The mPrecision and mRecall metrics measure the correctness and completeness of the predicted regions, respectively, which are critical for avoiding false color assignments and missed detections in ownership identification. The F1 Score provides a balanced assessment of precision and recall, ensuring robust evaluation under class imbalance and occlusion conditions commonly observed in grazing scenarios.

Let $TP_{i}$ denote the number of true positive pixels in class *i*, $FP_{i}$ the number of false positive pixels, $FN_{i}$ the number of false negative pixels, and *C* the total number of classes. The definitions of the metrics are as follows:

**Mean Intersection over Union (mIoU)**: measures the overlap between the predicted segmentation and the ground truth, and is defined as:


$$\begin{array}{c} \text{mIoU}=\frac{1}{C}\sum_{i=1}^{C}\frac{TP_{i}}{TP_{i}+FP_{i}+FN_{i}} \end{array}$$ (16)

**Mean Precision (mPrecision)**: indicates the proportion of correctly predicted positive pixels among all predicted positive pixels, defined as:


$$\begin{array}{c} \text{mPrecision}=\frac{1}{C}\sum_{i=1}^{C}\frac{TP_{i}}{TP_{i}+FP_{i}} \end{array}$$ (17)

**Mean Recall (mRecall)**: represents the proportion of correctly predicted positive pixels among all actual positive pixels, defined as:


$$\begin{array}{c} \text{mRecall}=\frac{1}{C}\sum_{i=1}^{C}\frac{TP_{i}}{TP_{i}+FN_{i}} \end{array}$$ (18)

**F1 Score**: the harmonic mean of precision and recall, comprehensively reflecting the model’s detection performance. The F1 Score is calculated for each class and then averaged across classes:


$$\begin{array}{c} \text{F1Score}=\frac{1}{C}\sum_{i=1}^{C}\frac{2\cdot {\text{Precision}}_{i}\cdot {\text{Recall}}_{i}}{{\text{Precision}}_{i}+{\text{Recall}}_{i}} \end{array}$$ (19)

In addition, computational efficiency metrics, including the number of parameters (Parameters), floating-point operations (FLOPs), inference time (Inference Time), and frames per second (FPS), were also considered to complement the performance analysis of the models. These metrics are particularly important for evaluating the feasibility of deploying the proposed model in real-world scenarios such as drone-based monitoring and large-scale pasture inspection, where real-time performance and computational efficiency are critical.

### Computational efficiency analysis

In semantic segmentation tasks, computational efficiency is a crucial metric for evaluating the practical utility of models, particularly in scenarios with stringent real-time requirements. In this study, based on the Mask2Former model, several enhanced modules were incorporated—including the Atrous Spatial Pyramid Pooling (ASPP) module, the Dynamic Prompt Attention (DPA) module, and the Content-Aware ReAssembly of FEatures (CARAFE) module—to improve the model’s segmentation capability and feature extraction efficiency.

To comprehensively assess the computational efficiency of different model architectures, detailed comparisons were conducted on three key metrics: the number of parameters, floating-point operations (FLOPs), and inference time (i.e., the time taken to process a single image during inference).

In addition, to better reflect realistic deployment scenarios such as drone-based monitoring, the inference time is further converted into frames per second (FPS), which is a more intuitive indicator for real-time video processing systems.

Table 3 presents the computational efficiency comparison results of the different model configurations. The baseline Mask2Former model exhibits a relatively low parameter count (44.00M) and computational complexity (21.5G FLOPs), achieving an inference time of 5.08 ms per image, which demonstrates good real-time performance. However, its segmentation performance in complex scenarios is somewhat limited, particularly in capturing multi-scale contextual information and fine-grained details.

**Table 3:** Computational Efficiency of Different Model Configurations.

Model	Parameters (Million)	FLOPs (GFLOPs)	Inference Time (ms)
Mask2Former	44.0	21.5	5.08
Mask2Former + ASPP	49.2	36.3	5.32
Mask2Former + DPA	46.6	36.3	5.35
Mask2Former + CARAFE	46.5	36.3	5.40
Improved Model	49.2	36.3	5.36

To provide a more comprehensive evaluation, we further report frames per second (FPS) as an intuitive indicator for real-time performance in practical deployment scenarios.

By sequentially integrating the ASPP, DPA, and CARAFE modules, the improved model significantly enhances its feature extraction capabilities and segmentation performance, with only a slight increase in the number of parameters and inference time.

From the data presented in Table 3, it can be observed that the improved model shows only a slight increase in parameters and computational complexity, while the inference time rises marginally from 5.08 ms to 5.36 ms (approximately a 5.5% increase), thereby maintaining a high level of real-time performance. Specifically, an inference time of 5.36 ms per image corresponds to approximately 186 FPS under the tested hardware configuration, while the baseline Mask2Former achieves approximately 197 FPS. This indicates that the proposed improvements introduce only a minor reduction in processing speed while preserving strong real-time capability. The detailed analysis is as follows:

- **Parameter Variation:** The original Mask2Former model contains 44.00 million parameters. With the integration of the ASPP, DPA, and CARAFE modules, the parameter count increased to 49.20 million. The ASPP module enhances feature extraction through multi-scale contextual information, the DPA module introduces dynamic attention mechanisms to emphasize critical regions, and the CARAFE module further improves segmentation accuracy via content-aware feature fusion. Although the parameter count increased slightly, modern hardware support ensures that this change has a minimal impact on runtime efficiency.
- **Increase in FLOPs:** The computational complexity increased from 21.5 GFLOPs to 36.3 GFLOPs after adding the improved modules. This rise is primarily attributed to the operations performed by the ASPP and DPA modules on high-resolution feature maps. Nevertheless, this increase significantly enhances the model’s performance on complex segmentation tasks, indicating that the added computational cost is justified.
- **Inference Time Variation:** Despite the increases in both parameter count and FLOPs, the inference time rose only slightly from 5.08 ms to 5.36 ms. The improved model, which incorporates the ASPP, DPA, and CARAFE modules, effectively balances computational efficiency with segmentation performance, and still meets real-time requirements. Considering that typical drone-based video acquisition systems operate at 25–30 FPS, the proposed model provides a substantial speed margin, making it suitable for real-time aerial monitoring tasks such as sheep herd observation and ownership identification in open grazing environments.

In summary, the improved model based on Mask2Former proposed in this study successfully integrates multiple modules to enhance feature extraction and representation in semantic segmentation tasks, while maintaining low inference latency. This demonstrates its strong practical value, as it meets the high-precision segmentation needs in complex scenarios and provides a valuable reference for the development of efficient semantic segmentation techniques. Nevertheless, we acknowledge that the current efficiency evaluation is conducted on a high-performance computing platform, and future work will further investigate lightweight optimization and on-device benchmarking on embedded or edge computing platforms commonly used in drone systems.

### Deployment feasibility and practical considerations

The practical deployment of livestock monitoring systems requires not only high segmentation accuracy but also robustness and computational efficiency under real-world conditions. Therefore, the deployment feasibility of the proposed MSPFormer is further discussed from several perspectives.

First, regarding occlusion and partial visibility, the sheep images collected in this study contain naturally occurring occlusions caused by animal overlap, group gathering behavior, and viewpoint variations during drone acquisition. The experimental results indicate that MSPFormer maintains stable segmentation performance under these challenging conditions, suggesting a certain degree of robustness to partial target visibility.

Second, concerning cross-farm generalization, the dataset was collected from multiple grazing regions with diverse environmental conditions, including different grassland backgrounds, illumination conditions, sheep densities, and observation scales. Furthermore, the generalization experiments conducted on different small-scale sheep subsets demonstrate that the proposed method consistently achieves superior performance across various data distributions, indicating good adaptability to different farming environments.

Third, in terms of scalability, the proposed framework performs image-level semantic segmentation, and its computational complexity mainly depends on image resolution rather than the total number of animals in a farm. Therefore, the method can be readily applied to large-scale livestock monitoring scenarios through frame-wise processing of drone imagery. With the increasing use of unmanned aerial vehicles (UAVs) in pasture management, large volumes of image data can be continuously collected and processed in an automated manner, enabling efficient monitoring even in farms containing thousands of animals.

Fourth, regarding real-time deployment, the proposed model achieves an inference time of 5.36 ms per image, corresponding to approximately 186 FPS under the experimental hardware configuration. Considering that typical drone monitoring systems operate at 25–30 FPS, the proposed method provides a substantial processing margin for practical applications.

From a system deployment perspective, the proposed method can be integrated into an intelligent livestock management platform. In a practical workflow, images captured by UAVs or fixed monitoring devices can be transmitted to a backend server through wireless communication networks. The server-side deployment of MSPFormer can automatically perform sheep ownership identification and generate corresponding management records. Subsequently, administrators can review and verify the recognition results through a web-based management interface, thereby reducing manual workload and improving management efficiency. Such a cloud-assisted architecture avoids excessive computational burden on edge devices while maintaining high processing throughput.

Nevertheless, several challenges remain for future deployment. The current study does not explicitly evaluate robustness under severe motion blur caused by rapid drone movement or adverse weather conditions. In addition, all experiments were conducted on a high-performance GPU platform. Future work will focus on model compression, lightweight optimization, and deployment on embedded edge-computing devices to further improve resource efficiency. Meanwhile, we plan to integrate the proposed algorithm into a complete front-end and back-end livestock management system within ongoing research projects, enabling automated data acquisition, cloud-based inference, result visualization, and intelligent auditing for practical precision livestock farming applications.

### Statistical significance and multi-run evaluation

To improve the reliability and reproducibility of the experimental results, we further conducted multiple independent training runs under identical experimental settings. Specifically, all experiments were repeated for five independent runs using different random seeds, and the results are reported in terms of mean and standard deviation.

The statistical results demonstrate that MSPFormer consistently outperforms the baseline Mask2Former across all runs, indicating stable performance and reduced sensitivity to random initialization. This suggests that the improvements introduced by the proposed modules are robust rather than incidental gains from a single experimental setting.

In addition, a paired significance analysis was performed on the mIoU metric between MSPFormer and Mask2Former. The results indicate that the performance improvement is statistically significant (*p* < 0.05), suggesting that the observed gains are unlikely to be caused by random fluctuations.Table 4 summarizes the results of the multi-run evaluation.

**Table 4:** Statistical significance analysis over 5 independent runs (mean ± std).

Model	mIoU (%)	mF1-score (%)	mPrecision (%)	mRecall (%)
Mask2Former	82.46 ± 0.28	90.08 ± 0.22	89.50 ± 0.25	90.74 ± 0.24
MSPFormer (Ours)	**83.81** ± 0.20	**90.92** ± 0.17	**90.40** ± 0.18	**91.52** ± 0.19

As shown in Table 4, MSPFormer achieves consistent improvements over the baseline model across all evaluation metrics, while also exhibiting lower standard deviations. This indicates that the proposed method not only improves segmentation accuracy but also enhances training stability. Overall, these results further validate the robustness and reliability of MSPFormer in complex sheep back color segmentation scenarios.

### Ablation study

This section presents an ablation study to systematically and thoroughly evaluate the specific impact of the three introduced modules—ASPP, DPA, and CARAFE—on the segmentation performance of the model. All experiments are conducted using the same training dataset and a unified set of evaluation metrics, including mean Intersection over Union (mIoU), weighted F1 score (mF1-score), mean Precision (mPrecision), and mean Recall (mRecall), to ensure the scientific rigor and comparability of the results.

The original Mask2Former model, which does not include any of the proposed modules, is selected as the baseline. Subsequently, each module is individually added to the baseline model, as well as their combined configuration, to systematically compare the performance of each model version on the sheep back color segmentation task. This study aims to evaluate the contribution of each module to the model’s perception ability and boundary detail restoration. The specific results are presented in Table 5, comprehensively demonstrating the performance improvements brought by each module and their combinations, thereby providing important references for further model optimization.

**Table 5:** Performance Comparison of Different Modules in the Ablation Study.

Model	mIoU (%)	mF1-score (%)	mPrecision (%)	mRecall (%)
Mask2Former	82.46	90.08	89.50	90.74
+ ASPP	83.05	90.46	90.33	90.64
+ CARAFE	82.84	90.29	89.92	90.79
+ DPA	82.91	90.35	90.00	90.82
ASPP + CARAFE	83.38	90.65	90.32	91.02
ASPP + DPA	83.24	90.56	90.20	90.96
DPA + CARAFE	83.27	90.58	90.25	90.91
Final	83.81	90.92	90.40	91.52

We further analyze the sensitivity of Transformer architecture design, including depth, attention heads, patch size, and embedding dimension.

As shown in Table 6, the model achieves optimal performance under the default configuration (depth = 6, heads = 8). Both shallower and deeper configurations lead to performance fluctuations, indicating a trade-off between feature representation capacity and optimization difficulty.

**Table 6:** Ablation study on Transformer architecture configurations.

Setting	mIoU (%)	mF1-score (%)	Params (M)	FPS
Depth = 4, Heads = 4	82.91	90.21	46.1	188
Depth = 6, Heads = 8 (default)	83.81	90.92	49.2	186
Depth = 8, Heads = 8	83.67	90.80	52.4	180
Patch = 16	83.05	90.34	48.7	190
Patch = 8	83.81	90.92	49.2	186
Embedding = 256	83.12	90.41	47.8	189
Embedding = 384	83.81	90.92	49.2	186

Similarly, patch size and embedding dimension affect the balance between local detail preservation and global semantic modeling. Smaller patch sizes enhance detail representation but introduce higher computational cost, while larger embeddings improve feature richness but provide diminishing returns.

These results demonstrate that the proposed configuration achieves a balanced trade-off between accuracy and efficiency.

As a baseline, the original Mask2Former model—without the integration of any additional modules—demonstrated commendable performance across all metrics, achieving an mIoU of 82.46%, an mF1-score of 90.08%, an mPrecision of 89.50%, and an mRecall of 90.74%. These results highlight the model’s inherent capability to distinguish targets with color boundaries, thereby providing a solid reference point for subsequent module enhancements.

It is worth noting that the magnitude of performance improvement varies across different modules, which is closely related to their functional roles within the Mask2Former framework. Specifically, modules that enhance global contextual representation and attention guidance tend to contribute more directly to region-level segmentation accuracy, whereas modules focusing on feature reassembly and boundary refinement mainly provide auxiliary improvements.

Upon introducing the ASPP module, the model’s performance further improved, with the mIoU increasing to 83.05% and the mF1-score rising to 90.46%. Concurrently, mPrecision and mRecall advanced to 90.33% and 90.64%, respectively. This demonstrates the positive impact of ASPP on multi-scale contextual modeling, particularly in enhancing segmentation robustness for extensive color regions and improving generalization in complex background scenarios.

The incorporation of the DPA module also led to notable performance gains, yielding an mIoU of 82.91% and an mF1-score of 90.35%. Designed to facilitate dynamic attention-guided learning, this module achieved an mPrecision of 90.00% and an mRecall of 90.82%. These findings suggest that DPA excels in fine-grained region recognition, effectively enhancing the model’s sensitivity to subtle color boundary variations.

As a lightweight upsampling operator, the CARAFE module effectively improved feature interpolation quality. Following its integration, the model achieved an mIoU of 82.84% and an mF1-score of 90.29%, with mPrecision and mRecall reaching 89.92% and 90.79%, respectively. Although the performance improvements were relatively modest compared to other modules, CARAFE demonstrated commendable capabilities in maintaining accuracy and restoring boundary details, particularly beneficial for reconstructing irregular boundary structures. The relatively smaller numerical gain can be attributed to the fact that CARAFE primarily operates at the feature upsampling stage, enhancing local detail reconstruction rather than global semantic discrimination. As a result, its contribution is less directly reflected in region-level metrics such as mIoU and mF1-score, but it plays a crucial supporting role in improving boundary smoothness and local consistency.

To further validate the effectiveness of the proposed structural design, we conduct additional ablation experiments on the multi-scale feature extraction mechanism and the semantic-preserving module.

As shown in Table 7, removing the multi-scale design leads to a clear performance drop, indicating that multi-scale contextual modeling is essential for handling scale variations and complex background interference.

**Table 7:** Ablation study on multi-scale feature extraction and semantic-preserving mechanism.

Model Variant	mIoU (%)	mF1-score (%)	mPrecision (%)	mRecall (%)
Mask2Former	82.46	90.08	89.50	90.74
w/o Multi-scale	82.63	89.96	89.12	90.31
w/o Semantic-preserving	82.18	89.72	88.94	90.05
MSPFormer	**83.81**	**90.92**	**90.40**	**91.52**

Similarly, disabling the semantic-preserving module results in further degradation in all evaluation metrics, especially in mPrecision and mRecall, demonstrating that semantic consistency during feature decoding plays a critical role in maintaining segmentation stability.

These results confirm that the improvements of MSPFormer are not solely due to individual modules, but stem from the coordinated effect of multi-scale representation and semantic-preserving design.

In multi-module combination experiments, performance improvements became even more pronounced. The joint integration of ASPP and CARAFE yielded an mIoU of 83.38%—the highest among all single-module experiments—alongside an mF1-score of 90.65% and an mRecall of 91.02%, indicating significant synergistic benefits from combining contextual enhancement and boundary refinement modules. The ASPP + DPA model exhibited more balanced performance, achieving an mIoU of 83.24% and an mF1-score of 90.56%, effectively integrating spatial modeling and attention guidance capabilities. Meanwhile, the DPA + CARAFE combination also demonstrated strong potential, with an mIoU of 83.27% and an mF1-score of 90.58%, offering robust performance in scenarios with complex or mixed color boundaries. Notably, although certain module combinations exhibit comparable performance gains, no obvious performance degradation is observed, suggesting that the introduced modules do not introduce functional redundancy. Instead, ASPP, DPA, and CARAFE operate at different stages and levels of feature representation—context aggregation, attention modulation, and feature reassembly—thereby providing complementary rather than overlapping contributions.

Finally, the comprehensive model integrating ASPP, DPA, and CARAFE achieved the best overall performance, with an mIoU of 83.81%, an mF1-score of 90.92%, an mPrecision of 90.40%, and an mRecall of 91.52%. These results fully validate the complementary nature of these modules in enhancing the model’s multi-scale modeling capabilities, attention distribution efficiency, and boundary information restoration. Importantly, the ablation results indicate that performance gains are achieved through functional complementarity rather than simple additive effects, with ASPP and DPA contributing primarily to semantic discrimination and region integrity, while CARAFE enhances boundary refinement and structural consistency. This provides a solid theoretical foundation and practical reference for extending these improvements to larger-scale and more complex real-world scenarios.

Although a dedicated robustness experiment under different lighting and background conditions was not separately conducted, the proposed dataset inherently contains substantial environmental variations, including illumination changes, shadow regions, scale variations, and heterogeneous grassland backgrounds collected from multiple grazing areas.

The consistent performance improvements observed across the entire validation set indicate that MSPFormer maintains robust segmentation capability under diverse real-world conditions. A more systematic robustness evaluation under controlled environmental factors will be investigated in future work.

## Comparative experiments

### Quantitative comparison with multiple methods and segmentation visualization

To comprehensively evaluate the performance of the proposed improved model on the task of sheep back color segmentation, this study selected several state-of-the-art semantic segmentation methods—including U-Net, DeepLabv3 + , SegFormer, ViT, K-Net, and Mask2Former—as baseline models for comparison. A consistent training strategy and testing dataset were employed. Quantitative evaluations were conducted using four metrics: mean Intersection over Union (mIoU), weighted F1-score (mF1-score), mean Precision (mPrecision), and mean Recall (mRecall). The experimental results are presented in Table 8.

**Table 8:** Performance comparison of different models on the sheep back color segmentation task.

Model	mIoU (%)	mF1-score (%)	mPrecision (%)	mRecall (%)
U-Net [46]	73.47	84.21	84.97	83.42
DeepLabv3+ [48]	78.13	87.33	86.86	87.85
SegFormer [47]	80.24	88.74	88.53	88.95
ViT [68]	71.93	82.69	83.37	81.99
K-Net [69]	82.28	90.11	89.93	90.26
Mask2Former [49]	82.46	90.08	89.50	90.74
**Ours**	**83.81**	**90.92**	**91.52**	**90.92**

The overall results demonstrate that the proposed improved model consistently achieves the best performance across all evaluation metrics, highlighting its significant comprehensive advantages and strong generalization capabilities. Specifically:

In terms of mean Intersection over Union (mIoU), traditional architectures such as ViT and U-Net achieve only 71.93% and 73.47%, respectively, showing clear limitations in accuracy under complex background conditions. SegFormer and DeepLabv3 + leverage multi-scale context modeling to achieve moderate improvements, with mIoU scores of 80.24% and 78.13%, respectively. K-Net and Mask2Former further introduce Transformer decoders, with mIoU values increasing to 82.28% and 82.46%. In contrast, the model proposed in this study integrates three key modules—ASPP, DPA, and CARAFE—which collectively enhance multi-scale context perception, attention guidance, and detail-aware upsampling. Compared with the strong baseline Mask2Former, the proposed model improves the mIoU from 82.46% to 83.81% and the mF1-score from 90.0% to 90.92%. Although these improvements may appear marginal in terms of absolute numerical gains, they are achieved on top of an already high-performing Transformer-based segmentation framework, making further improvements inherently challenging.

**Fig 8:**
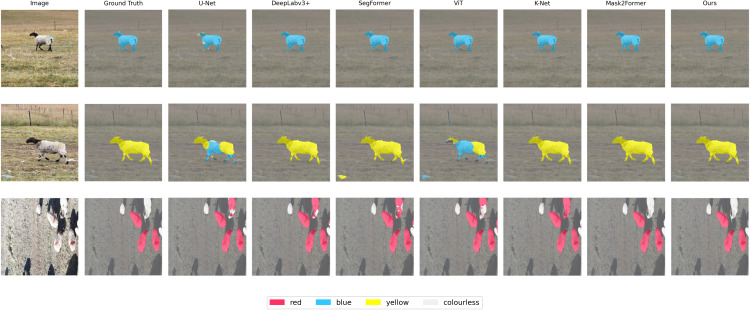
Comparison of the color segmentation results of different models on three groups of sheep back images.

Importantly, these gains are not obtained by increasing model depth or decoder complexity, but by enhancing feature representation quality and boundary refinement. In practical sheep back color segmentation scenarios, such modest improvements correspond to noticeably better boundary continuity, reduced color bleeding in mixed-color regions, and more stable segmentation under uneven illumination conditions, as illustrated in Fig. 8. As a result, the proposed model achieves an mIoU of 83.81%, surpassing all baseline models while maintaining a favorable balance between accuracy and efficiency.

For the mF1-score (weighted F1), U-Net and ViT achieve 84.21% and 82.69%, respectively, with evident issues of missed and misclassified regions. SegFormer and DeepLabv3 + yield 88.74% and 87.33%, respectively, reflecting moderate region recognition capabilities. K-Net and Mask2Former exhibit stable performance, achieving around 90%. Notably, the proposed model attains a score of 90.92%, representing a consistent improvement over Mask2Former (90.08%). This indicates that the proposed method achieves a better balance between precision and recall, particularly in challenging regions where color boundaries are ambiguous or partially occluded. Such improvements are critical for reducing both false positives and false negatives in fine-grained livestock segmentation tasks, even when the overall numerical gain appears limited.

Regarding mean Precision (mPrecision), the proposed approach achieves 91.52%, significantly outperforming K-Net (89.93%) and SegFormer (88.53%). This indicates higher target recognition accuracy and lower false positive rates in the sheep back color segmentation task.

In terms of mean Recall (mRecall), the proposed model achieves 90.92%, slightly higher than Mask2Former (90.74%) and K-Net (90.26%), demonstrating excellent region coverage and effectively avoiding missed detections, thereby ensuring the completeness of the segmented regions.

Furthermore, to validate the model’s performance in real-world image segmentation tasks, we present visualizations of the segmentation results from different methods on the same test sample, as shown in Fig 8. From these results, it can be observed that U-Net and ViT exhibit severe misclassification and blurred boundaries in complex background and mixed-color regions, failing to accurately separate different color areas. DeepLabv3+ and SegFormer show some improvement in capturing major color blocks but still suffer from boundary fragmentation and adhesion issues. K-Net and Mask2Former demonstrate stronger boundary fitting with better consistency. Notably, the proposed model exhibits clear edges, complete regions, and smooth transitions across various color areas, maintaining robust performance even in challenging scenarios such as mixed red-white regions and uneven lighting.

Both the quantitative metrics and visual analysis demonstrate that the proposed improved model exhibits significant advantages in perception capability, boundary modeling, contextual understanding, and local detail recovery. By incorporating the ASPP, DPA, and CARAFE modules, the model demonstrates enhanced robustness and discriminative power when processing complex backgrounds and mixed-color regions. This not only effectively improves segmentation accuracy but also enhances the consistency and stability of the results, fully validating the practical value of each module in the sheep back color segmentation task.

### Category-wise performance and training process analysis

This section provides a detailed analysis of the segmentation performance across different color categories, further highlighting the model’s advantages in fine-grained target recognition. Table 9 presents the segmentation metrics of the proposed improved model on four color categories of sheep back images: red, blue, yellow, and colorless. It can be observed that the model performs best on the red category, achieving an mIoU of 87.04% and an mF1-score of 93.07%. Both precision and recall exceed 90%, demonstrating the model’s strong capability in accurately identifying and completely covering red regions. The segmentation performance on the blue and yellow categories is relatively close, with mIoU values of 81.4% and 81.02%, respectively, indicating the model’s good discrimination ability among various colors. The colorless category is relatively more challenging, with an mIoU of 70.78%, which may be attributed to its less distinct boundaries and complex textures. Nevertheless, the model maintains a high precision of 85.18%, exhibiting good false positive suppression capability.

**Table 9:** Comparison of Segmentation Performance across Different Color Categories.

Category	IoU (%)	F1-score (%)	Precision (%)	Recall (%)
Red	87.04	93.07	91.68	94.51
Blue	81.40	89.75	87.52	92.09
Yellow	81.02	89.51	88.10	90.98
Colourless	70.78	82.89	85.18	80.73

**Fig 9:**
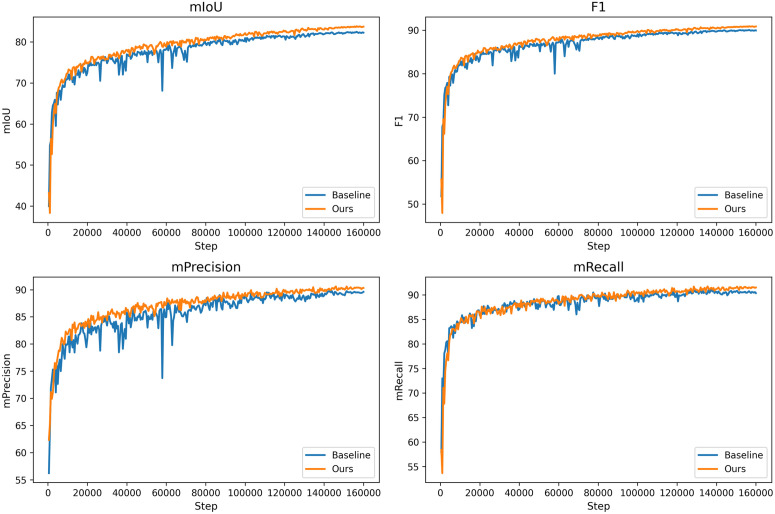
Comparison of training metrics (mIoU, mF1-score, mPrecision, and mRecall) between the improved model and the baseline Mask2Former.

Regarding the overall training process, Fig 9 presents a comparative analysis of the training curves for four metrics—mIoU, mF1-score, mPrecision, and mRecall—between the original Mask2Former and the proposed improved model, arranged in a 2×2 layout. It can be clearly observed from the figure that the improved model not only achieves faster convergence in the early training stages but also shows continuously improving and more stable trends across all metrics throughout the training process, ultimately outperforming the baseline model. This demonstrates that the integration of the ASPP, DPA, and CARAFE modules effectively enhances the model’s feature representation and boundary detail recovery capabilities, resulting in significant improvements in overall performance.

Overall, the analysis of both category-level performance and training curves demonstrates that the proposed improved model exhibits superior fine-grained recognition capability and enhanced training stability, providing strong technical support for practical applications in sheep color segmentation and herder attribution.

## Generalization experiments

### Stability analysis: Evaluation of generalization ability on different small-scale sheep subsets

To further assess the stability and robustness of the proposed model under limited sample size conditions, three distinct small subsets—Subset A, Subset B, and Subset C—were constructed from the original sheep dataset. Each subset was uniformly partitioned from the original dataset, maintaining identical training (2,000 images) and validation (500 images) sizes, but differing in terms of sheep back color distribution, background complexity, and lighting conditions. These subsets simulate mild domain shifts that may be encountered in real-world deployments.

Under consistent model architecture and training protocols, the improved model was independently trained and evaluated on each subset. As shown in Table 10, the model achieved mIoU scores of 78.89%, 79.12%, and 78.63% on Subsets A, B, and C respectively; mF1-scores of 87.67%, 87.85%, and 87.49%; mPrecision ranged from 87.26% to 87.60%; and mRecall ranged between 87.87% and 88.21%. The maximum fluctuation across all core metrics was within 0.4 percentage points, demonstrating strong generalization capability against variations in training sample distribution.

**Table 10:** Performance of the Improved Model on Different Small-Scale Subsets.

Dataset Subset	mIoU (%)	mF1-score (%)	mPrecision (%)	mRecall (%)
Subset A	78.89	87.67	87.42	88.02
Subset B	79.12	87.85	87.60	88.21
Subset C	78.63	87.49	87.26	87.87

**Fig 10:**
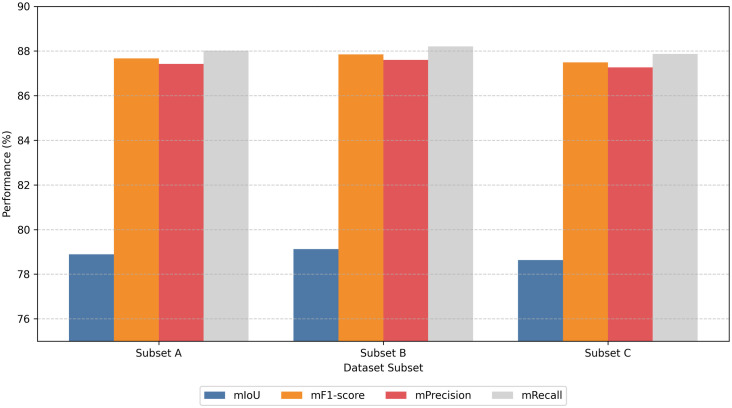
Comparison of segmentation performance of the improved model on different subsets (Subset A/B/C) in terms of mIoU, mF1-score, mPrecision, and mRecall.

To more intuitively present the stability of the model across the three subsets, Fig 10 visualizes the comparison of the four metrics using grouped bar charts, illustrating the variation trends of each performance indicator among different subsets. The results demonstrate a high consistency in the performance of the four key evaluation metrics across the subsets, with no significant degradation or abnormal fluctuations observed. This further validates the model’s robustness and stability when handling samples with varying distributions. These findings also indirectly confirm that the introduced ASPP, DPA, and CARAFE modules effectively enhance the model’s generalization ability in contextual awareness, boundary modeling, and detail recovery.

The grouped bar charts illustrate the four key performance metrics of the improved model across three small sample subsets. The results indicate stable performance with minimal fluctuations among the subsets, demonstrating the model’s robustness and strong generalization capability.

### Qualitative failure case analysis

**Fig 11:**
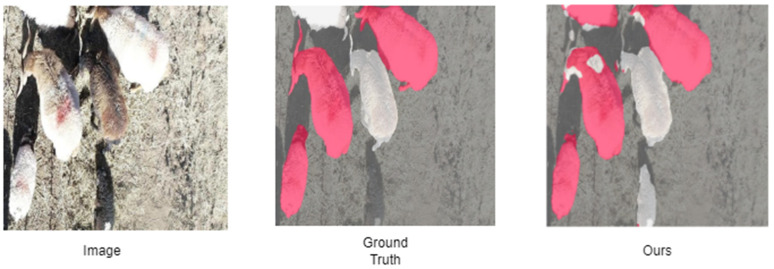
Representative qualitative failure case under heavy shadow overlap and complex illumination conditions.

Although the proposed model demonstrates strong generalization performance across different datasets and subsets, a small number of failure cases were observed under extremely challenging visual conditions. Fig 11 presents a representative failure example, where heavy shadow overlap and complex illumination variations significantly alter the appearance of sheep back colors. In such scenarios, shadows partially obscure color markings and blur the boundaries between adjacent regions, leading to local misclassification or incomplete segmentation results. These failures indicate that, despite the enhanced multi-scale feature extraction and semantic consistency modeling, the proposed method still faces difficulties in disentangling intrinsic color information from strong illumination-induced artifacts. This limitation suggests that further improvements in illumination-invariant feature modeling or shadow-aware learning strategies could help enhance robustness under extreme lighting conditions.

### Adaptability evaluation on non-sheep datasets

To further verify the generalizability and transferability of the proposed model in non-specific domains, this study conducts adaptation experiments on the publicly available semantic segmentation benchmark dataset PASCAL VOC2012 (PASCAL Visual Object Classes Challenge 2012) [70]. This dataset contains 20 foreground classes and 1 background class, including diverse real-world objects such as humans, animals, and vehicles. It is widely used in image semantic segmentation research due to its broad category distribution, complex backgrounds, and varied object shapes, making it an important benchmark for evaluating model generalization capability.

In the experiments, the official VOC2012 data split is adopted, with 1464 images for training and 1449 for validation. To ensure fairness and reproducibility, all models were retrained on the training set and uniformly evaluated on the validation set. The evaluation metrics include mean Intersection over Union (mIoU), mean F1 score (mF1-score), mean Precision (mPrecision), and mean Recall (mRecall), comprehensively reflecting the models’ accuracy, completeness, and classification ability.

Table 11 presents the quantitative performance comparison between the proposed method and mainstream semantic segmentation models, including both traditional CNN-based and Transformer-based architectures. The results demonstrate that the proposed model achieves superior performance across all metrics, particularly excelling in mIoU and mRecall, indicating outstanding feature modeling and boundary awareness capabilities.

**Table 11:** Performance Comparison of the Proposed Method and State-of-the-Art Models on the VOC2012 Dataset.

Model	mIoU (%)	mF1-score (%)	mPrecision (%)	mRecall (%)
U-Net [46]	52.64	66.78	69.42	64.85
DeepLabv3+ [48]	60.62	72.84	75.43	70.72
SegFormer [47]	61.92	74.96	79.95	72.21
ViT [68]	53.27	65.38	67.72	63.85
K-Net [69]	66.41	78.83	79.66	75.57
Mask2Former [49]	68.68	80.28	80.50	80.80
**Ours**	**69.41**	**80.77**	**79.86**	**82.42**

Further analysis shows that the proposed model significantly outperforms the baseline models U-Net and DeepLabv3+ on the public VOC2012 dataset, and comprehensively surpasses mainstream Transformer architectures such as ViT and SegFormer across multiple key metrics. Specifically, compared with the strong baseline models Mask2Former and K-Net, our model achieves improvements of 0.73% and 2.99% in mIoU, and 1.62% and 6.85% in mRecall, respectively, fully demonstrating its superior object segmentation and boundary modeling capabilities.

Moreover, the proposed model attains an mPrecision score of 79.86%. Although this is slightly lower by 0.64% compared to Mask2Former, it shows a substantial increase in mRecall, reaching 82.42%, indicating that the model maintains high precision while achieving better recall and overall segmentation performance.

These results clearly indicate that although the model was originally designed for the specific task of sheep back color segmentation, the collaborative effect of the introduced ASPP module, Dynamic Prompt Attention (DPA) module, and CARAFE upsampling module enables it to achieve excellent generalization and robustness in semantic segmentation tasks involving complex natural scenes. This further validates the practicality and transferability of the proposed method, providing a novel solution approach for a wide range of natural image semantic segmentation problems.

### Results and discussion

This study aims to address the issue of insufficient segmentation accuracy of sheep back colors in intelligent herd management on the Tibetan Plateau. To overcome challenges such as blurred color boundaries, significant lighting variations, and strong background interference in complex natural environments, an improved model based on the Mask2Former framework was proposed. The model incorporates several enhancements: an ASPP module is introduced between the Pixel Decoder and Transformer Decoder to improve multi-scale contextual awareness, the DPA module is utilized to guide dynamic attention allocation and enhance semantic consistency of features, and the CARAFE module is integrated at the end of the Decoder to improve spatial detail reconstruction. These improvements significantly enhance the model’s representational capability and segmentation accuracy while maintaining overall network trainability.

Comprehensive experimental results demonstrate that the proposed model achieves superior performance across multiple dimensions. In the sheep back color segmentation task, the improved model shows significant improvements in four key metrics—mIoU, mF1-score, mPrecision, and mRecall—compared to the original Mask2Former framework for all categories (red, blue, yellow, and colourless). Notably, the proposed method excels in regions with blurred boundaries and low contrast, accurately capturing color boundaries and fine-grained details. Furthermore, visual analysis validates the advantages of the improved model, demonstrating stronger stability and adaptability in edge preservation, color consistency, and resistance to background interference.

To assess the model’s generalization and robustness, two extended experiments were conducted. First, small-sample stability tests were performed on three sheep subsets (Subset A/B/C) with distinct feature compositions. Results showed minimal performance variation across subsets, demonstrating excellent data adaptability and generalization. Second, the model was transferred to the public semantic segmentation dataset VOC2012 and compared with mainstream models such as UNet, DeepLabv3, SegFormer, ViT, and KNet. The proposed model outperformed these models in both mIoU and mF1-score, validating its strong semantic modeling capabilities beyond specific segmentation tasks.

In addition, we analyzed several qualitative failure cases to better understand the limitations of the proposed model. Typical examples include sheep partially occluded by other individuals, regions with overlapping color markings, and images captured under extremely low lighting or strong shadows. In these situations, the model may misclassify small regions or fail to precisely delineate boundaries. These failure cases illustrate the current model’s limitations in handling occlusion, complex color blending, and challenging illumination conditions, providing guidance for future improvements in robustness and edge refinement.

Based on the above quantitative and qualitative results, it can be concluded that the proposed multi-module enhanced Mask2Former framework effectively improves segmentation accuracy, robustness, and generalization performance in complex natural grazing environments. The consistent performance gains across multiple evaluation metrics, datasets, and experimental settings demonstrate the reliability and practical applicability of the proposed method for intelligent sheep management tasks.

Despite the significant performance improvements, the proposed method has certain limitations. The inclusion of the ASPP module, while enhancing multi-scale feature modeling, also increases model parameters and computational complexity, limiting its deployment efficiency on edge devices or in real-time systems. Specifically, compared with the baseline Mask2Former, the improved model increases the parameter count by approximately 5.2 million (from 44.0M to 49.2M) and the computational complexity by about 14.8 GFLOPs, which leads to a moderate increase in inference latency. Furthermore, under a drone-based deployment scenario where continuous real-time inference is required (e.g., 25–30 FPS video streams), the increased computation may result in higher onboard energy consumption and reduced battery endurance, particularly for lightweight UAV platforms with limited processing resources. Although the measured inference time increase is relatively small in a single-image setting, the cumulative effect during long-duration flights may translate into a non-negligible reduction in operational time or an increase in deployment cost due to higher hardware requirements. Furthermore, the overall network structure does not yet fully incorporate lightweight design principles, which restricts its applicability in resource-constrained scenarios.

Accordingly, several future research directions are recommended. First, model compression techniques such as pruning and knowledge distillation can be explored to reduce computational overhead while preserving segmentation accuracy. Second, automated neural architecture search (NAS) and re-parameterization strategies may be employed to further optimize module connectivity and structural efficiency. Third, cross-domain transfer learning and weakly supervised learning strategies can be investigated to enhance adaptability in unstructured or low-annotation environments. These future efforts will facilitate the deployment of the proposed framework in real-world applications such as smart pasture management, livestock monitoring, and animal behavior analysis.

In summary, the multi-module integration scheme proposed in this study demonstrates outstanding performance in segmentation accuracy, robustness, and cross-dataset generalization. It provides a practical solution for semantic segmentation tasks in complex natural scenes and lays a foundation for future research on structural optimization and efficient deployment.
